# Myometrial Microbiome in Uterine Fibroids: Functional Dysbiosis Versus Limited Taxonomic Change

**DOI:** 10.3390/biology15171498

**Published:** 2026-09-02

**Authors:** Sara Bertorello, Francesco Cei, Simone Baldi, Flavia Sorbi, Francesco La Torre, Gianluca Bartolucci, Silvia Vannuccini, Massimiliano Fambrini, Elena Niccolai, Felice Petraglia, Amedeo Amedei

**Affiliations:** 1 Department of Experimental and Clinical Medicine, University of Florence, 50139 Florence, Italy; sara.bertorello@unifi.it (S.B.);; 2 Department of Experimental and Clinical Biomedical Sciences “Mario Serio”, University of Florence, 50134 Florence, Italy; 3 Department of Neuroscience, Psychology, Drug Research and Child Health NEUROFARBA, University of Florence, 50139 Florence, Italy; 4 Network of Immunity in Infection, Malignancy and Autoimmunity (NIIMA), Universal Scientific Education and Research Network (USERN), 50139 Florence, Italy

**Keywords:** uterine fibroids, myometrium, microbiome, free fatty acids, cytokines

## Abstract

**Background:** Uterine fibroids (UFs) are the most common benign tumors in women of reproductive age and are associated with abnormal uterine bleeding, infertility, and impaired implantation and endometrial receptivity. However, the contribution of the myometrial microbiome (MM) to fibroid biology and the broader uterine environment remains poorly understood. **Materials and Methods:** We characterized the paired MM of UF tissue and adjacent healthy myometrium (HM) from 21 women undergoing surgery and integrated serum free fatty acid (FFA) and cytokine profiling. **Results:** Taxonomic composition and microbial diversity were highly similar between tissues, with no differentially abundant taxa after multiple-testing correction. In contrast, PICRUSt2-based functional prediction indicated tissue-specific differences in microbial metabolic potential: UF tissue showed higher predicted representation of pathways related to L-tyrosine biosynthesis and aromatic amine degradation, whereas HM showed higher predicted representation of coenzyme A and arginine biosynthesis pathways. Patients also exhibited distinct circulating FFA profiles compared with healthy women, and integrative analyses identified phenotype-specific associations among microbial taxa, FFAs, and inflammatory mediators, particularly in women with heavy menstrual bleeding or specific fibroid localizations. **Conclusions:** These findings suggest that UFs are associated with potential functional remodeling of the MM rather than major taxonomic alterations, which may contribute to changes in the uterine environment relevant to implantation, endometrial receptivity, and reproductive health.

## Simple Summary

Uterine fibroids are very common non-cancerous growths of the uterus that can cause heavy menstrual bleeding, pelvic pain, infertility and pregnancy complications. They may also impair the uterus’ ability to support embryo implantation and successful pregnancy, although the underlying biological mechanisms remain poorly understood. In this study, we examined the bacteria naturally present in fibroid tissue and in nearby healthy uterine muscle from the same women. We also measured blood fatty acids, which can be influenced by both host physiology and intestinal bacteria, together with inflammatory molecules. We found that the bacterial communities were largely similar in healthy and fibroid tissues, but their predicted biological functions differed. Women with uterine fibroids also showed peculiar blood fatty acid profiles compared with healthy women, and the relationships among bacteria, fatty acids and inflammatory molecules varied according to clinical features such as heavy menstrual bleeding and fibroid location. These findings suggest that changes in bacterial function, rather than major shifts in bacterial composition, may contribute to an altered uterine environment that could affect implantation, endometrial receptivity and reproductive health. Understanding these interactions may support the development of new biomarkers and future strategies to improve fertility care.

## 1. Introduction

Uterine fibroids (UFs) are the most common benign tumors of the female reproductive tract, affecting up to 50–70% of women of reproductive age [1,2]. In approximately one third of cases, UFs become clinically relevant, causing abnormal uterine bleeding, mainly heavy menstrual bleeding, pelvic symptoms and reproductive dysfunction, with a substantial impact on quality of life and a need for tailored medical, surgical and interventional management strategies [3]. Beyond their symptomatic burden, UFs have been associated with impaired reproductive outcomes, including reduced implantation rates, increased miscarriage risk, and altered pregnancy success, particularly when lesions are located close to or distort the endometrial cavity, as with submucosal and selected intramural fibroids [4,5]. UFs arise from monoclonal proliferations of smooth muscle cells within the myometrium and are largely driven by estrogen and progesterone [6,7]. However, despite their strong hormonal responsiveness, UF pathogenesis remains only partially understood, with additional non-hormonal determinants implicated, including genetic susceptibility, obesity, lifestyle and diet [1,2].

Collectively, these factors may influence host–microbiome interactions, with the gut microbiome (GM) potentially contributing to UF biology through altered production of microbial metabolites, including short-chain fatty acids (SCFAs) and, to a lesser extent, medium- (MCFAs) and long-chain fatty acids (LCFAs), which are involved in immune regulation, epithelial barrier integrity and hormonal signaling [8,9].

Among these metabolites, SCFAs are particularly relevant to the reproductive context. In detail, butyric and propionic acids have been implicated in the regulation of immune cell functions, including uterine natural killer cell activity and regulatory T-cell differentiation at the maternal–fetal interface, processes that contribute to endometrial receptivity and successful implantation [10].

Additionally, SCFAs act through G-protein-coupled receptors, including GPR41 and GPR43, which are expressed in reproductive tissues and may link microbial metabolite availability to local immune and epithelial signaling [11].

Notably, microbiome-mediated metabolic effects are shaped by the surrounding niche, including oxygen tension, substrate availability and local inflammatory conditions, contributing to substantial inter-individual variability in their biological impact [12].

Over the last two decades, the human microbiome has attracted increasing attention for its potential role in reproductive health. Growing evidence suggests that UFs may influence the uterine microenvironment beyond the fibroid lesion itself, affecting biological processes relevant to fertility, including uterine contractility, endometrial receptivity, implantation-related pathways and local inflammatory responses [5,13]. Given the estrogen-dependent nature of UF development [14], increasing interest has focused on the estrobolome, the collection of microorganisms involved in estrogen metabolism, as a potential mediator of endocrine and immune interactions. Although current evidence remains largely indirect or derived from related conditions (e.g., breast cancer or conceptual models of the uterine niche) [15,16], recent studies have reported alterations in GM composition among patients with fibroids, including shifts in Proteobacteria and Firmicutes/Bacteroidota balance, suggesting a potential systemic microbial influence on UF biology [17].

More recently, the traditional concept of the uterus as a sterile environment has been challenged. Chen et al. demonstrated the presence of distinct microbial communities throughout the female reproductive tract, including the endometrium [18]. Subsequent studies have described *Lactobacillus*-dominant endometrial profiles in healthy conditions, whereas increased microbial diversity has been reported in pathological states such as endometriosis, polyps and potentially UFs [19]. Moreover, endometrial microbial composition has been associated with reproductive outcomes in infertile patients, with *Lactobacillus*-dominant profiles generally correlating with improved implantation, pregnancy and live birth rates, supporting a possible role of the uterine microbiome in endometrial receptivity and reproductive success [20]. However, whether UF-associated microbial alterations contribute to impaired reproductive outcomes remains largely unexplored, representing a critical gap in understanding the biological mechanisms linking UFs, uterine microbial ecology and fertility [5]. Most previous studies have relied on fecal or vaginal sampling, providing limited insight into the microbial environment of uterine tissue. Furthermore, taxonomic profiling alone may fail to capture functional alterations, reflecting the increasingly recognized dissociation between microbial composition and biological activity [21]. The present study, for the first time, integrated 16S rRNA gene sequencing of paired UF tissue and adjacent healthy myometrium (HM) with profiling of serum free fatty acids (FFAs) and inflammatory cytokines. By combining local myometrial microbiome characterization with systemic metabolic and inflammatory readouts, this study aims to elucidate the contribution of the uterine microbiome to UF pathophysiology and to investigate whether microbiome-associated functional signatures can provide mechanistic insights into the altered uterine environment potentially involved in fibroid-associated reproductive dysfunction, including impaired implantation and altered endometrial receptivity.

## 2. Materials and Methods

### 2.1. Sample Collection

A total of 42 fresh uterine tissue samples were collected from 21 patients undergoing surgical treatment for UFs at Careggi University Hospital, Florence, Italy.

Endometrial samples were collected before surgery using a Pipelle de Cornier (Prodimed, Neuilly-en-Thelle, France) endometrial sampler. At the beginning of surgery, before any manipulation of the uterus, paired myometrial specimens were collected from the UF lesion (*n* = 21) and from macroscopically healthy myometrium (HM) distant from the fibroid in the same patient. The HM specimens (*n* = 21) were collected separately from the UF tissue and were not derived from the endometrium. Endometrial, UF, and HM tissues were sampled independently using dedicated sterile instruments to minimize the risk of cross-contamination and were processed separately for downstream DNA extraction and 16S rRNA gene sequencing.

The clinical and demographic characteristics of the study population are reported in Table 1. Median age was 48 years (interquartile range [IQR]: 14, Q1 = 39, Q3 = 53). For each patient, relevant clinical information was collected, mostly as categorical variables (presence/absence of findings or symptoms). Recorded items included heavy menstrual bleeding (HMB), adenomyosis, and presence of subserosal fibroids, as well as data on infertility, endometriosis, pelvic pain, multiple fibromatosis, and intramural myoma. Data on BMI, dietary habits, hormonal status or treatment, antibiotic exposure, and concomitant medication use were not systematically available and could therefore could not be included as covariates in the microbiome, serum FFA, or cytokine analyses.

All tissue specimens were immediately frozen at −80 °C upon collection and stored until further processing. Peripheral blood samples were obtained to assess the serum FFA levels; following centrifugation, serum was aliquoted and stored at −20 °C until gas chromatography–mass spectrometry (GC-MS) analysis. Additionally, 21 healthy female controls were age-matched to UF patients for serum FFA analysis.

All participants provided written informed consent prior to inclusion in the study. The study protocol was approved by the local ethics committee and conducted in accordance with the principles of the Declaration of Helsinki.

### 2.2. Microbiome Characterization

Genomic DNA was extracted from tissue biopsies using the DNeasy Blood & Tissue Kit (Qiagen, Hilden, Germany), following the manufacturer’s protocol. Given the low-biomass nature of myometrial tissue, a negative control consisting of an empty tube containing the same extraction reagents but no biological material was processed in parallel with the tissue samples. The negative control underwent DNA extraction, 16S rRNA gene amplification, library preparation, and sequencing alongside the biological samples, allowing potential contamination introduced during sample processing and subsequent sequencing procedures to be monitored. DNA concentration and purity were assessed using both the NanoDrop ND-1000 spectrophotometer and the Qubit Fluorometer (Thermo Fisher Scientific, Waltham, MA, USA). DNA samples were then stored at −20 °C until further analysis.

Amplicons targeting the V3–V4 hypervariable region of the bacterial 16S rRNA gene were generated using the primers 341F (5′-CCTACGGGNGGCWGCAG-3′) and 785R (5′-GACTACHVGGGTATCTAATCC-3′) and sequenced in paired-end mode (2 × 250 bp) on the Illumina NovaSeq platform by IGA Technology Services (Udine, Italy), according to the Illumina 16S Metagenomic Sequencing Library Preparation protocol.

Demultiplexed paired-end reads were imported into QIIME2 (version 2021.4) using the CasavaOneEightSingleLanePerSampleDirFmt format. Primer sequences were removed using the QIIME2 Cutadapt (v.3.4) trim-paired plugin, specifying the forward primer CCTACGGGNGGCWGCAG and reverse primer GACTACHVGGGTATCTAATCC; reads lacking the expected primer sequences were discarded (--p-discard-untrimmed). Sequence quality was assessed after primer removal, and 100,000 reads were randomly sampled for quality-summary visualization. DADA2 (v.1.18) was subsequently used for paired-end denoising, quality filtering, read merging, chimera removal, and inference of amplicon sequence variants (ASVs), with forward and reverse reads truncated at 224 and 223 bp, respectively (--p-trunc-len-f 224, --p-trunc-len-r 223). To eliminate potential host contamination, resulting amplicon sequence variants (ASVs) were aligned to the GRCh38 human reference genome using Bowtie2 (v. 2.4.4), and matching sequences were discarded.

Remaining high-quality reads were imported into QIIME2 for taxonomic classification using a Scikit-learn naive Bayes classifier (v.0.24.1), trained on the SILVA 138 reference database specific to the V3–V4 region. ASVs with a mean relative abundance below 0.005% were excluded to minimize the influence of potential contaminants.

Sequences assigned to the *Chloroplast* genus were removed, in accordance with previous literature [22]. Since myometrial tissue is a low-biomass sample type, potential contaminant sequences were minimized through host-read removal, exclusion of very low-abundance ASVs, removal of chloroplast-assigned sequences, and evaluation of the negative extraction control. These contamination-control steps were considered when interpreting microbial profiles to reduce the possibility that detected bacterial features reflected technical artifacts rather than true biological variation.

### 2.3. Evaluation of Circulating Metabolites and Inflammatory Mediators

Serum FFA levels, namely circulating SCFAs (acetic, propionic, butyric, isobutyric, 2-methylbutyric, isovaleric and valeric acids), MCFAs (hexanoic, isohexanoic, octanoic, decanoic and dodecanoic acids) and LCFAs (tetradecanoic, hexadecanoic, and octadecanoic acids), were quantified using GC–MS. Analyses were performed on an Agilent GC–MS system (Agilent Technologies, Santa Clara, CA, USA) consisting of a 5890-gas chromatograph, 5971 single-quadrupole mass spectrometer, and 7673 autosampler. Standard curves for quantification were prepared as previously described [23].

Serum IL-1β, IL-12p70, IL-18, IL-10, IL-6, TNFα, CCL2, osteoprotegerin (OPG), and LPS-binding protein (LBP) were quantified (pg/mL) using the ELLA Simple Plex microfluidic immunoassay platform (ProteinSimple/Bio-Techne, San Jose, CA, USA) according to the manufacturer’s instructions. Blood was collected in serum separator tubes, allowed to clot for 30 min at room temperature, and centrifuged at 1500–2000 g for 10 min at 4 °C; serum aliquots were then stored at −80 °C and thawed on ice and diluted as recommended before analysis. Each sample was assayed in on-cartridge technical triplicate, with an eight-point calibration curve using a 4-parameter logistic model.

### 2.4. Statistical Analyses

Statistical analyses of bacterial communities were performed in R (v.4.2.2; R Foundation for Statistical Computing, Vienna, Austria) with the following packages: phyloseq (v.1.42.0), vegan (v.2.6-4), DESeq2 (v.1.38.3), ggplot2 (v.3.2.4), Hmisc (v.5.2.3), ggpubr (v.0.6.0), and additional supporting libraries. No a priori sample-size or power calculation was performed, as the study cohort was determined by the availability of eligible patients and paired tissue specimens during the study period. Accordingly, analyses of the overall cohort, and mainly subgroup analyses, should be considered exploratory. To assess sequencing depth adequacy, rarefaction curves were generated using the rarecurve function. Alpha diversity was evaluated at the genus level based on observed richness, the Shannon diversity index, and Pielou’s evenness index, calculated as E = S/log(R), where S is the Shannon index and R is observed genus richness. Differences in alpha diversity indices between groups were tested using the Wilcoxon signed-rank test, with *p*-values < 0.05 considered statistically significant.

Beta diversity was analyzed using principal coordinate analysis (PCoA) on Hellinger-transformed genus-level abundance data. Statistical significance of clustering was assessed by PERMANOVA (9999 permutations) on Hellinger distance matrices. Differential abundance analyses at various taxonomic ranks were conducted using the DESeq2 algorithm on raw count data; taxa with adjusted *p*-values (Benjamini–Hochberg correction) < 0.05 were considered significantly different, and features with a grand mean count < 100 were excluded to reduce background noise. Predicted functional profiles were inferred using PICRUSt2 (v.2.4.2) with the EPA-ng algorithm; MetaCyc pathway profiles were therefore interpreted as inferred functional potential based on 16S rRNA gene composition, rather than as directly measured microbial gene content, transcriptional activity, or metabolite production. Differences in predicted MetaCyc pathway representation between groups were explored using LEfSe (v.1.1.2, Linear Discriminant Analysis Effect Size); these analyses were considered exploratory and hypothesis-generating.

Concentrations of FFAs and cytokines were analyzed using the Wilcoxon–Mann–Whitney test or the Kruskal–Wallis test, followed by Dunn’s post hoc test when appropriate. For individual FFA and cytokine comparisons, *p*-values were adjusted using the Benjamini–Hochberg false discovery rate (FDR) method when multiple comparisons were performed. Finally, Spearman’s rank correlations were calculated between the top 15 genera, cytokine levels, and FFA concentrations. For each tissue- and subgroup-specific correlation matrix, *p*-values were adjusted for multiple testing using the Benjamini–Hochberg FDR method; correlations with adjusted *p*-values (padj) < 0.05 were considered statistically significant. Given the limited sample size of the subgroup analyses, correlation results were considered exploratory and hypothesis-generating.

## 3. Results

### 3.1. Taxonomic Composition of the Myometrial Microbiome

#### 3.1.1. Overall Microbial Composition

**Figure 1:**
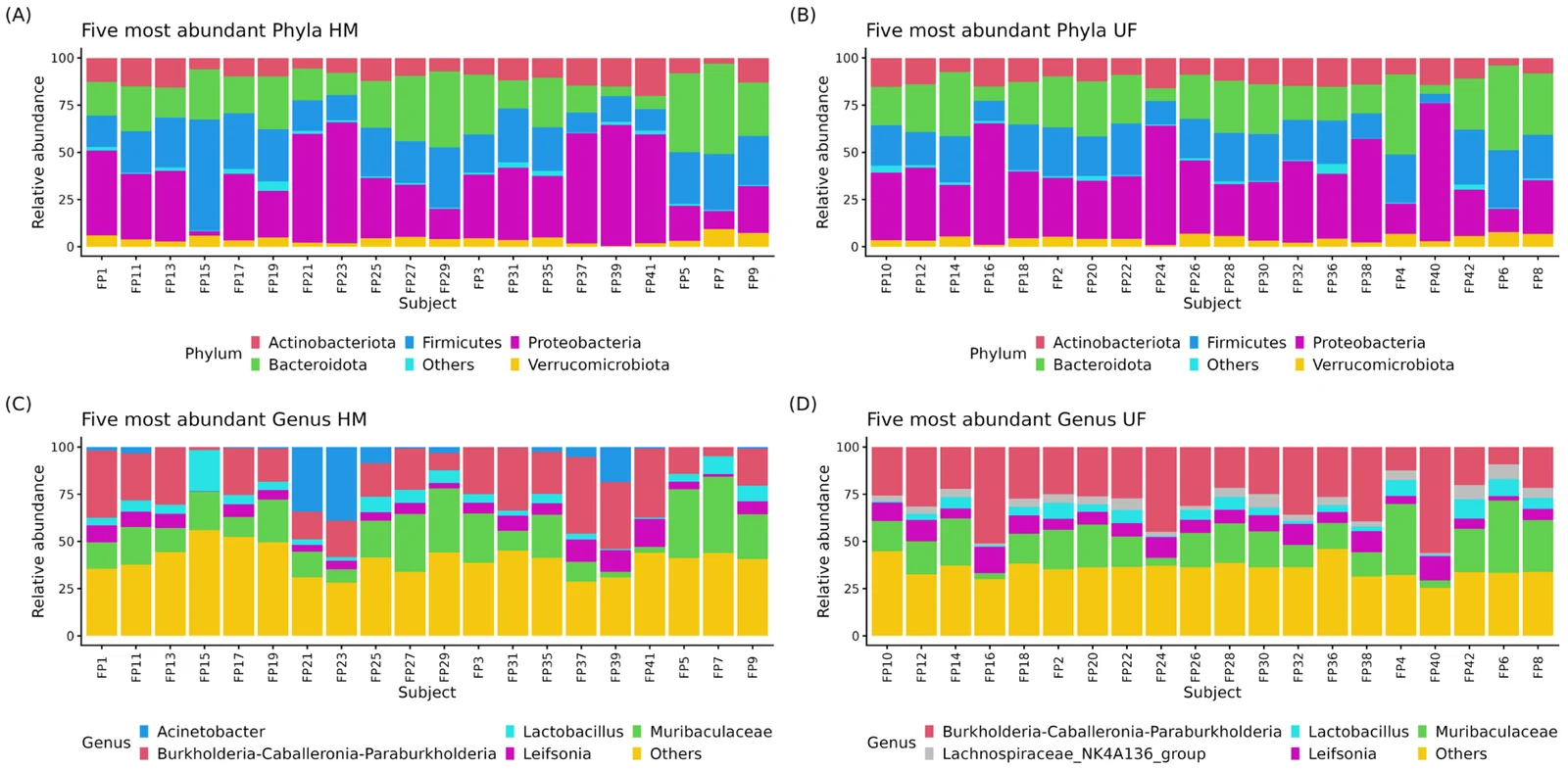
Microbial composition of adjacent HM and UF tissue. Stacked bar plots showing the average relative abundance of the top five phyla and genera in adjacent HM (**A**,**C**) and UF tissue (UF tissue (**B**,**D**)).

Sequencing generated a mean of 57,399 reads per HM sample and 79,385 reads per UF tissue sample, with mean filtered reads of 19,111 and 33,389, respectively. The negative extraction control processed in parallel with tissue samples yielded no retained reads after quality filtering, denoising, and downstream processing, indicating no detectable bacterial signal attributable to the extraction workflow. Both HM and UF tissue microbiomes were dominated by five predominant bacterial phyla: Proteobacteria, Firmicutes, Bacteroidota, Actinobacteriota, and Verrucomicrobiome (Figure 1A,B). At the genus level, the most abundant taxa in HM were *Burkholderia-Caballeronia-Paraburkholderia* (22.6%), *Muribaculaceae* (19.2%), *Leifsonia* (6.4%), *Acinetobacter* (6.0%), and *Lactobacillus* (5.6%) (Figure 1C).

The most represented genera in UF tissue samples were *Burkholderia-Caballeronia-Paraburkholderia* (29.0%), *Muribaculaceae* (18.5%), *Leifsonia* (8.1%), *Lactobacillus* (4.7%), and *Lachnospiraceae_NK4A136_group* (4.3%). The same predominant genera were also observed in HM samples, except that *Acinetobacter* replaced *Lachnospiraceae_NK4A136_group* (Figure 1D).

**Figure 2:**
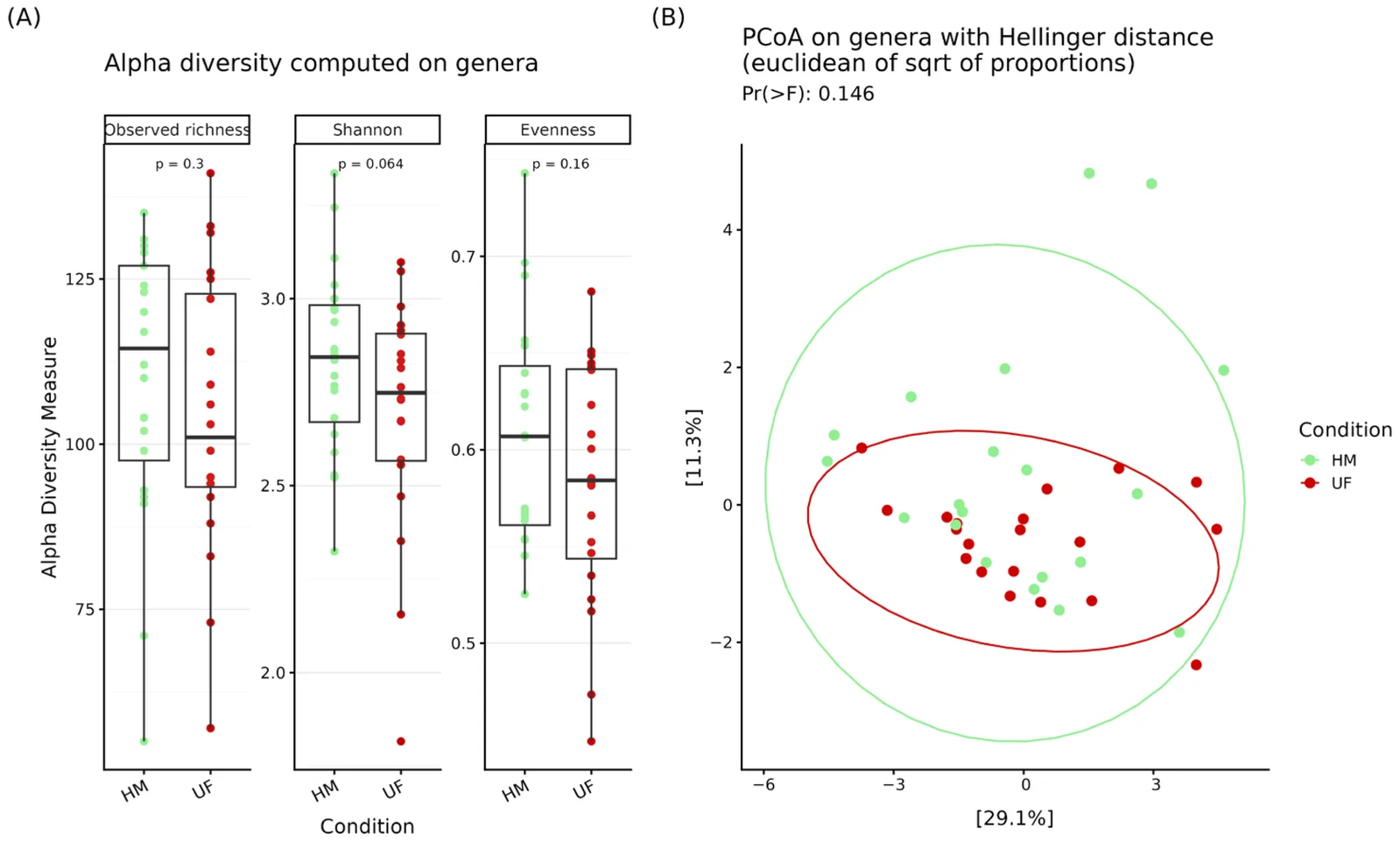
Alpha and beta diversity analysis of microbial communities in HM and UF tissue. (**A**) Box plots showing alpha diversity indices (observed ASV richness, Shannon index, and Pielou’s evenness) in HM and UF tissue samples. Statistical differences were assessed using the Wilcoxon test, with *p* < 0.05 considered statistically significant. (**B**) Principal coordinate analysis (PCoA) based on Hellinger distance of genus-level transformed abundance data, comparing microbial communities between HM and UF tissue samples.

When comparing tissue types directly, alpha diversity indices did not differ significantly between HM and UF tissue samples (Figure 2A). Similarly, beta diversity analysis using Hellinger-transformed abundances showed no distinct clustering between compartments (Figure 2B), and differential abundance testing (DESeq2) did not identify any taxa significantly enriched in either tissue type after correction for multiple testing. Overall, only minimal compositional differences were observed between diseased and adjacent healthy tissue.

#### 3.1.2. Microbiome Profiles According to Clinical Characteristics

Stratification by clinical characteristics revealed clearer patterns. Among patients with HMB, UF tissue samples showed significantly decreased microbial diversity, with lower Shannon diversity and evenness compared with non-HMB patients (Supplementary Figure S1). Notably, these differences were specific to the diseased tissue, as no significant diversity changes were observed in the matched HM samples. Finally, no significant differences in alpha or beta diversity were detected when UF tissue and HM samples were stratified by fibroid localization (subserosal vs non-subserosal; Supplementary Figure S2).

### 3.2. Functional Prediction Based on 16S rRNA Gene Data

#### 3.2.1. Differentially Predicted Metabolic Pathways

**Figure 3:**
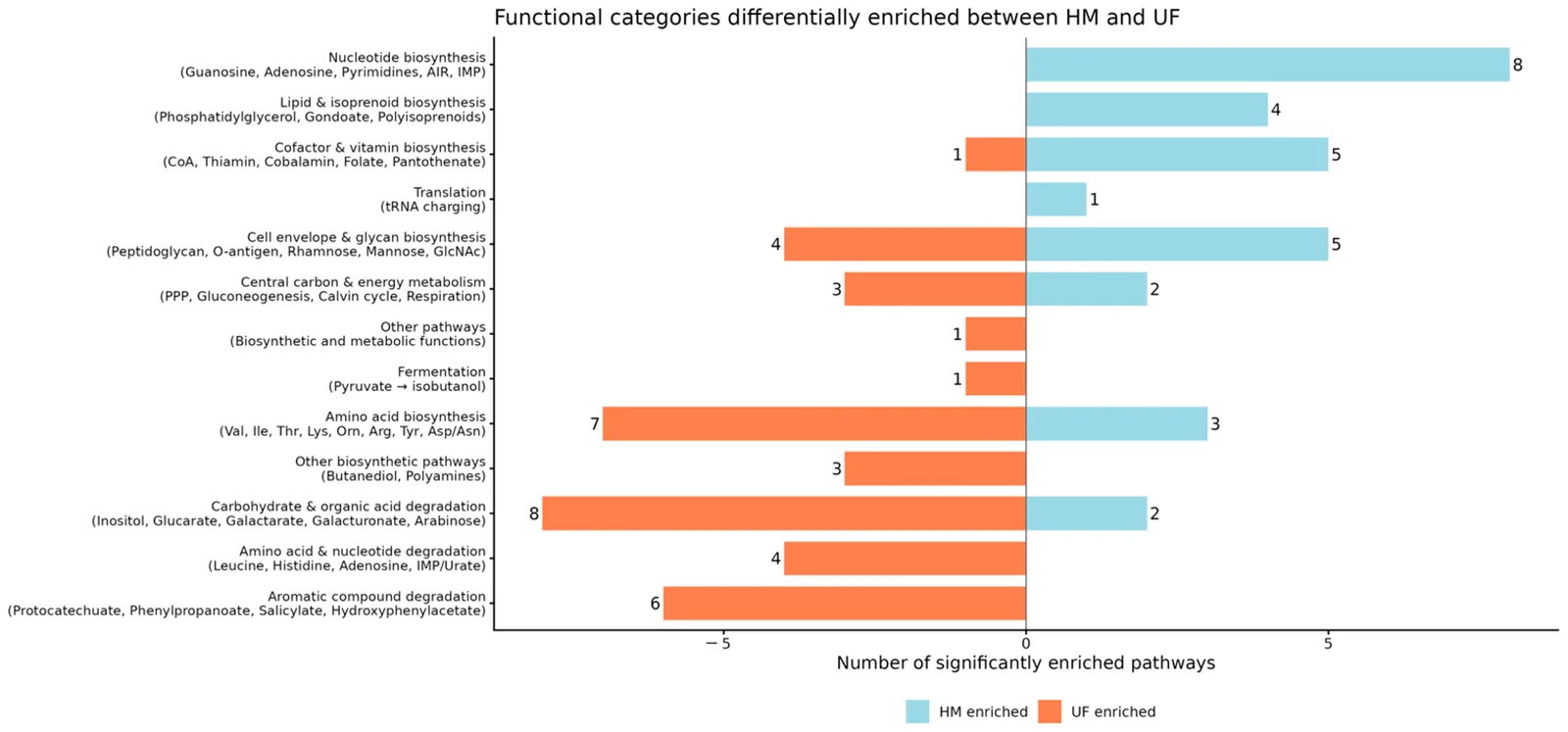
Predicted microbial functional pathways associated with HM and UF tissue. PICRUSt2-inferred MetaCyc pathways showing differential predicted representation between HM and UF tissue samples based on LEfSe analysis were grouped into broader functional categories to facilitate biological interpretation. Bars indicate the number of significantly enriched pathways within each functional category, with direction indicating enrichment in HM or UF samples. Representative pathway classes or metabolites included within each category are reported in parentheses. The complete list of individual MetaCyc pathways and corresponding LDA scores is provided in Supplementary Table S1. These results represent predicted functional potential inferred from 16S rRNA gene data and should not be interpreted as directly measured microbial functions.

PICRUSt2-based functional inference was used to explore predicted microbial pathway potential from 16S rRNA gene data. This analysis identified differences in 68 inferred MetaCyc pathways between UF tissue and HM, with 38 showing higher predicted representation in UF tissue and 30 showing higher predicted representation in HM. To facilitate biological interpretation, differentially represented pathways were grouped into broader functional categories in Figure 3, while the complete list of individual MetaCyc pathways and corresponding LDA scores is provided in Supplementary Table S1. Overall, HM showed a greater representation of pathways related to nucleotide, lipid and cofactor/vitamin biosynthesis, whereas UF tissue was characterized by a higher representation of pathways related to carbohydrate and organic acid degradation, aromatic compound degradation, and amino acid and nucleotide degradation. At the individual pathway level, coenzyme A and arginine biosynthesis showed higher predicted representation in HM, whereas UF tissue showed higher inferred representation of pathways related to L-tyrosine biosynthesis and aromatic amine degradation.

#### 3.2.2. Functional Profiles According to Clinical Characteristics

Grouping by clinical features revealed additional variation in PICRUSt2-inferred functional profiles. Patients with HMB showed differences in predicted pathway representation compared with non-HMB patients, and these patterns were observed in both UF tissue and HM. Similarly, pathway differences associated with fibroid localization (subserosal vs. non-subserosal) were mirrored across the two tissue compartments (Supplementary Tables S2 and S3). Thus, within the limits of PICRUSt2-based inference, these exploratory results suggest that clinical characteristics such as HMB status and fibroid localization may contribute to variation in predicted microbial functional profiles beyond tissue type alone.

### 3.3. Serum Cytokine and Free Fatty Acid Profiles

#### 3.3.1. Serum FFA Profiles in UF Patients and Healthy Controls

**Figure 4:**
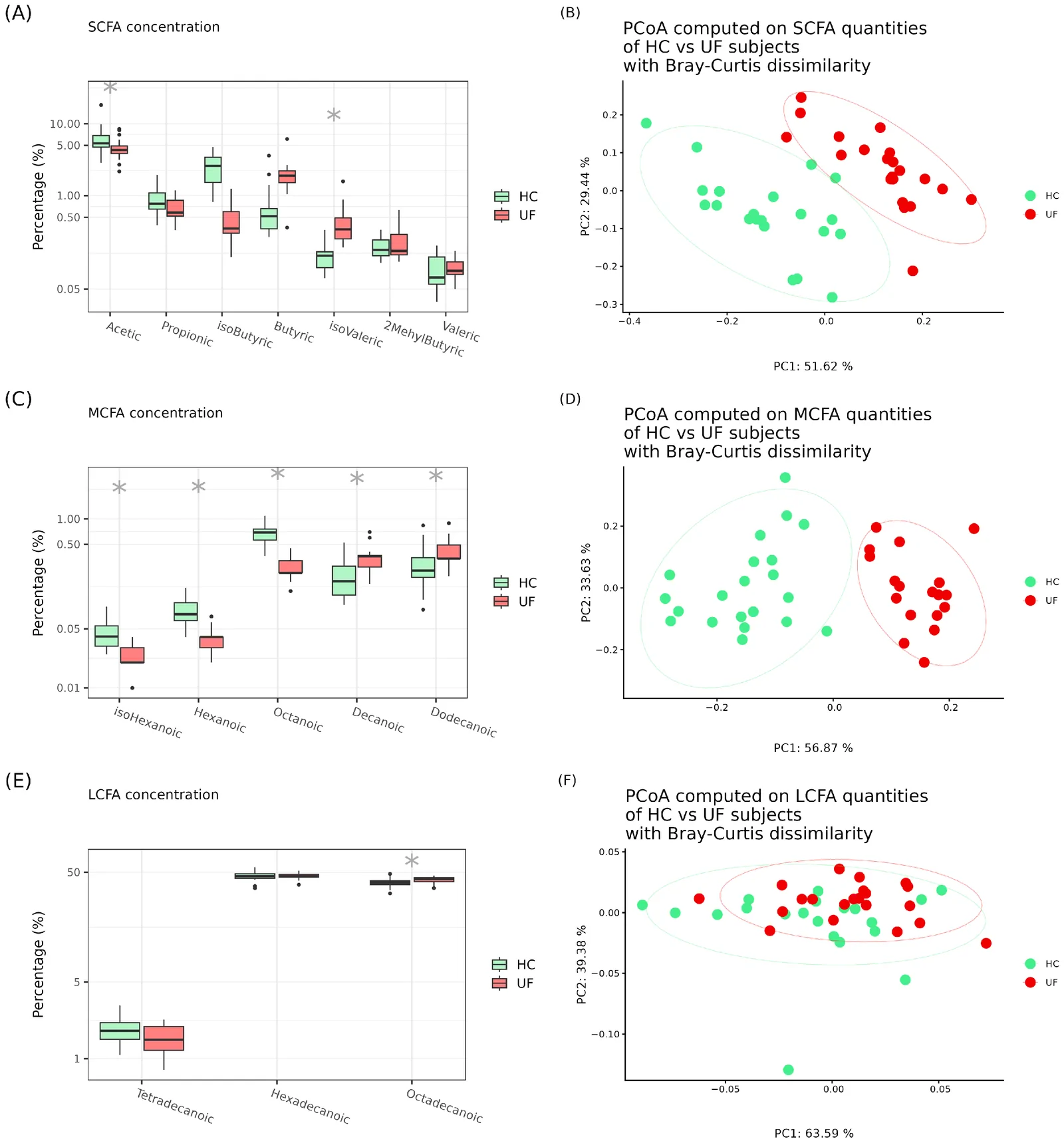
Serum FFA profiles differ between patients with UF and healthy controls. Box plots showing differences in the relative abundance of circulating SCFAs (**A**), MCFAs (**C**), and LCFAs (**E**) between UF patients and healthy controls. Statistical comparisons were performed using the Mann–Whitney test with Benjamini–Hochberg correction; asterisks (*) indicate adjusted p-values < 0.05 and black dots represent individual samples. Principal coordinate analysis (PCoA) based on the Bray–Curtis dissimilarity index showing differences in circulating SCFAs (**B**), MCFAs (**D**), and LCFAs (**F**) between UF patients and healthy female controls. Statistical significance was assessed using permutational multivariate analysis of variance (PERMANOVA).

To characterize the systemic immune-metabolic profile of UF patients and explore its relationship with local microbial features, we quantified circulating FFAs and cytokines. Comparison of the metabolic profile between UF patients and age-matched healthy female controls revealed marked alterations in patients’ serum FFAs. Multivariate analysis showed a clear separation between groups, with SCFAs and MCFAs differing substantially (PERMANOVA *p* = 0.003) (Figure 4,B,D,F). At the individual metabolite level, UF patients exhibited lower serum acetic (padj = 0.03) and isobutyric acids (padj < 0.0001), but higher butyric (padj < 0.0001) and isovaleric acids (padj = 0.009) compared with controls (Figure 4A). Among MCFAs, patients showed increased decanoic (padj = 0.001) and dodecanoic acids (padj = 0.004), and decreased isohexanoic (padj < 0.0001), hexanoic (padj < 0.0001), and octanoic acids (padj < 0.0001) (Figure 4C). Among LCFAs, octadecanoic acid was significantly elevated in UF patients (padj = 0.03) (Figure 4E).

Interestingly, serum FFA levels did not discriminate according to HMB status or fibroid localization (Supplementary Figures S3 and S4).

#### 3.3.2. Cytokine Profiles

Finally, we evaluated the systemic inflammatory profile by quantifying circulating cytokines and found that cytokine concentrations did not differ across clinical subgroups overall (Supplementary Tables S4 and S5). These findings suggest a relatively homogeneous systemic inflammatory profile in UF patients, regardless of clinical presentation.

### 3.4. Correlation and Integration of Serum Data with the Myometrial Microbiome

To investigate whether specific immune-metabolic–microbial interactions characterized distinct clinical subgroups, we performed an integrated correlation analysis combining the myometrial microbiome, circulating FFAs and cytokine levels. Our aim was to determine whether UF patients with HMB, or with different fibroid localizations, showed distinct association patterns between systemic metabolic markers and local microbial communities.

**Figure 5:**
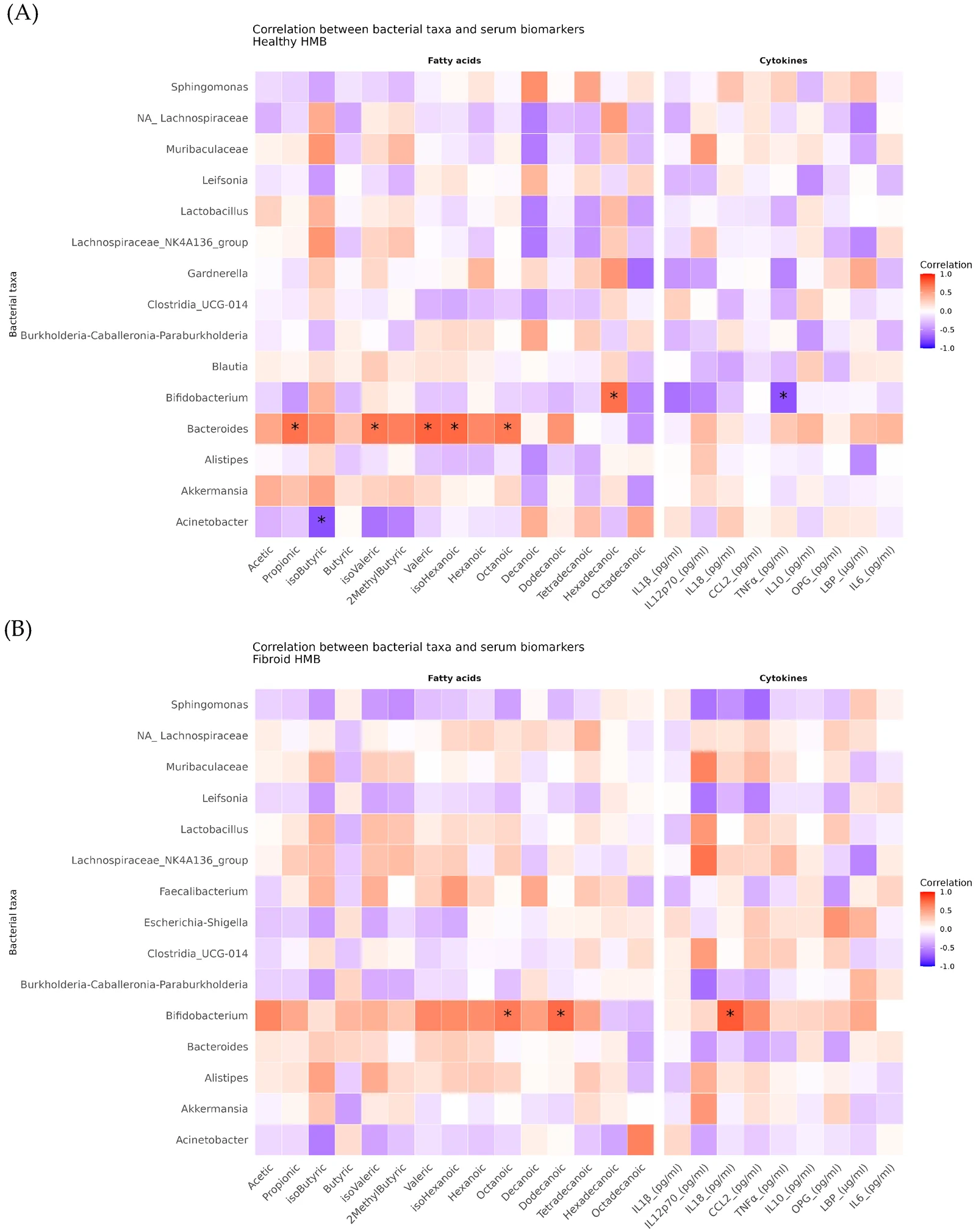
Correlation patterns between serum FFAs, inflammatory cytokines, and tissue microbiome in UF patients with HMB. Heatmaps of Spearman correlations between serum FFAs, circulating cytokines and tissue microbial taxa in UF patients with HMB. Panel (**A**) shows correlations for HM samples, whereas panel (**B**) shows correlations for UF tissue samples. Red shades indicate positive correlations, whereas blue shades indicate negative correlations; color intensity reflects the strength of the correlation. Correlations were computed using Spearman’s rank method. Asterisks (*) denote statistically significant correlations after multiple-testing correction (padj < 0.05).

Notably, in UF patients with HMB, integration of the three datasets revealed tissue-specific correlation profiles (Figure 5). In HM samples, *Bacteroides* showed significant positive correlations with several FFAs, including propionic (ρ = 0.70; padj = 0.03), isovaleric (ρ = 0.69; padj = 0.03), valeric (ρ = 0.77; padj = 0.01), isohexanoic (ρ = 0.74; padj = 0.01) and octanoic (ρ = 0.68; padj = 0.04) acids. *Acinetobacter* spp. correlated negatively with isobutyric acid (ρ = −0.75; padj = 0.01), whereas *Bifidobacterium* spp. was positively associated with hexadecanoic acid (ρ = 0.69; padj = 0.04) and negatively associated with TNFα (ρ = −0.76; padj = 0.02) (Figure 5A). In contrast, the UF tissue compartment displayed a different set of associations: *Bifidobacterium* spp. correlated positively with octanoic acid (ρ = 0.67; padj = 0.04), dodecanoic acid (ρ = 0.73; padj = 0.01) and IL-18 (ρ = 0.80; padj = 0.01) (Figure 5B).

These correlations were not observed in UF patients without HMB, suggesting that heavy menstrual bleeding may be associated with distinct microbiome–metabolite–cytokine interaction patterns in UFs (Supplementary Figure S5).

Finally, stratification by fibroid localization revealed additional subgroup-specific interactions. In UF patients without subserosal myoma, the healthy myometrial compartment displayed multiple significant positive correlations between *Bacteroides* spp. and several circulating FFAs, including acetic (ρ = 0.85; padj = 0.02), propionic (ρ = 0.83; padj = 0.02), isovaleric (ρ = 0.81; padj = 0.03), 2-methylbutyric (ρ = 0.86; padj = 0.009), valeric (ρ = 0.89; padj = 0.003) and octanoic acids (ρ = 0.81; padj = 0.02) (Supplementary Figures S6 and S7).

In the same compartment, *Gardnerella* spp. showed a significant anticorrelation with TNFα (ρ = −0.78; padj = 0.046). In the corresponding UF tissue, by contrast, we observed a more limited interaction pattern, characterized by a positive correlation between *Bifidobacterium* and tetradecanoic acid (ρ = 0.86; padj = 0.01).

Patients with subserosal fibroids, in contrast, showed a distinct correlation profile. In healthy myometrial tissue, *Faecalibacterium* spp. was positively associated with circulating LBP levels (ρ = 0.96; padj = 0.04), whereas in fibroid tissue, *Acinetobacter* spp. showed a significant anticorrelation with CCL2 (ρ = −0.95; padj = 0.01).

Overall, these exploratory findings suggest that fibroid localization may be associated with distinct immune-metabolic–microbial correlation patterns, potentially influencing the extent and nature of systemic–local interactions.

## 4. Discussion

The present study provides the first comprehensive characterization of the myometrial microbiome based on paired sampling of UF tissue and adjacent HM from the same patients. At the taxonomic level, the uterine parenchymal microbiome appeared remarkably stable: α- and β-diversity metrics were largely conserved between UF tissue and HM, and no taxa met the significance threshold for differential abundance following multiple testing adjustments. Nevertheless, specific genera showed differential representation, with *Acinetobacter* spp. among the most abundant taxa in HM and the *Lachnospiraceae NK4A136 group* spp. relatively enriched in UF tissue. These findings align with prior descriptions of the upper genital tract, including the endometrium, fallopian tubes and pouch of Douglas, where microbial communities are typically not *Lactobacillus*-dominant and *Acinetobacter* spp. is frequently detected [18,24]. These communities are therefore better interpreted as site-specific rather than “*Lactobacillus*-deficient”, consistent with the broader pattern of niche-specific variability across the uterine environment [18]. Moreover, the known sensitivity of endometrial microbiome profiles to sampling strategies, even within the same patient population [2], underscores the strength and novelty of our paired-tissue design.

While taxonomic differences were subtle, PICRUSt2-based functional inference indicated differences in predicted microbial pathway potential between tissue types. HM showed higher inferred representation of coenzyme A and arginine biosynthesis pathways, whereas UF tissue showed higher predicted representation of pathways related to L-tyrosine biosynthesis and aromatic amine degradation. The higher predicted representation of aromatic-amine-related pathways in UF tissue is of interest, given that microbial-derived aromatic metabolites and biogenic amines have been implicated in host signaling pathways, although their actual production within the myometrial environment remains to be established [25]. Notably, previous metabolomic studies in reproductive disorders have reported alterations in amino acid metabolism, including tyrosine-related pathways, suggesting that changes in aromatic amino acid metabolism may reflect broader inflammatory and metabolic adaptations, and may relate to impaired endometrial receptivity [26,27]. These observations suggest that predicted functional alterations may occur despite relative microbial compositional stability. However, PICRUSt2-based predictions require experimental validation, and the observed pathway differences should therefore be considered exploratory and hypothesis-generating.

Overall, these findings suggest potential shifts in microbial functional capacity in UF despite limited taxonomic differences; however, confirmation using direct functional approaches, such as shotgun metagenomics, metatranscriptomics, or targeted myometrial metabolomics, will be required [21,28,29].

These predicted functional changes may be biologically relevant in the context of the altered uterine microenvironment associated with fibroids, which has been proposed to involve tissue remodeling, mechanotransduction, inflammatory signaling and changes in endometrial receptivity. Within this framework, microbiome-associated predicted functional remodeling may represent an additional component influencing the local biological milieu that supports implantation and reproductive competence. Fibroid-associated reproductive dysfunction is likely multifactorial and may involve altered uterine contractility, implantation-related pathways, local inflammation and potentially changes in the vagino-uterine microbiome, although these mechanisms remain incompletely characterized [4,5,13].

These expected functional differences, observed despite relative compositional stability, resemble patterns seen in other pathological conditions, such as colorectal cancer, where modest changes in microbial structure can coexist with substantial functional remodeling [30,31]. Conversely, the higher predicted representation of coenzyme A and arginine biosynthesis pathways in HM may reflect preserved anabolic and nitric-oxide-related homeostatic processes.

Stratification by gynecological phenotype suggested that predicted functional profiles may vary across clinical subgroups. UF patients with HMB showed differences in PICRUSt2-inferred pathway representation compared with those without HMB, and subserosal fibroids were associated with predicted pathway configurations that differed from non-subserosal lesions. These exploratory findings suggest that inferred microbial functional potential may be influenced by the mechanical and hormonal microenvironment associated with different fibroid morphologies and clinical manifestations, rather than representing a common UF signature. Consistent with reports of context-dependent microbial functional profiles in reproductive and intestinal conditions [21,28], the myometrial functional landscape appears to mirror both tissue state and broader clinical phenotype.

At the systemic level, serum metabolomics revealed marked differences in circulating free fatty acids between UF patients and healthy female controls. Multivariate analyses showed a clear separation in SCFA, MCFA and LCFA profiles, while specific metabolites, including decreased acetic and isobutyric acids and increased butyric acid, isovaleric acid, and selected MCFAs and LCFAs, were significantly altered. Because circulating FFAs reflect the combined contribution of host lipid metabolism, dietary factors and, for selected SCFAs, microbial fermentation, these changes are best interpreted as a systemic metabolic signature associated with UFs rather than as a direct readout of the low-biomass uterine microbial community. Nonetheless, the observed pattern of FFA alterations is compatible with an imbalance in microbiome-derived metabolites capable of influencing immune regulation, endocrine signaling and myometrial physiology, including estrogen-sensitive pathways [8,9,10,11,32,33,34].

Serum cytokine levels were overall comparable across UF patient subgroups defined by clinical characteristics, indicating no detectable variation in systemic inflammatory status within the UF cohort. However, in an integrative framework, cytokines contributed meaningfully to context-specific association networks. In patients with UFs and HMB, HM samples showed positive correlations between *Bacteroides* spp. and multiple SCFAs and MCFAs, alongside anti-correlations involving *Acinetobacter* spp. with isobutyric acid and *Bifidobacterium* spp with TNFα; UF tissue samples, by contrast, were characterized by coordinate associations between *Bifidobacterium* spp., selected MCFAs and IL-18. Notably, these networks were not observed in non-HMB UF patients, suggesting that HMB may be associated with distinct microbiome–metabolite–cytokine interaction patterns. Distinct networks also emerged in patients stratified by fibroid localization, further highlighting the influence of anatomical phenotype on immunometabolic–microbial crosstalk.

Although exploratory, these findings support the hypothesis of a context-dependent microbiome–immune–lipid axis in uterine fibroids that warrants validation in larger independent cohorts. A better understanding of these interactions may provide novel insights into the mechanisms through which fibroids alter uterine function and contribute to reproductive impairment, including changes in implantation and endometrial receptivity. Future studies integrating microbiome, metabolomic and functional reproductive outcomes will be required to determine whether these signatures represent biomarkers of fibroid-associated reproductive dysfunction, or potential therapeutic targets [4,5,13].

## 5. Conclusions

In summary, our results suggest that the myometrial microbiome of paired HM and UF tissue specimens is largely taxonomically stable, whereas PICRUSt2-based analyses indicate potential differences in predicted microbial functional potential according to tissue status and clinical phenotype. Thus, microbial differences in UFs may be reflected in changes in predicted functional potential despite limited taxonomic variation. Although preliminary, these explorative findings support the emerging and relevant concept that disease-associated host–microbiome interactions may involve functional changes beyond conventional taxonomic dysbiosis [12,21].

Notably, these results may contribute to a better understanding of how UFs modify the local uterine environment beyond the fibroid lesion itself. Fibroid-associated reproductive impairment is well documented as a multifactorial phenomenon involving alterations in uterine contractility, endometrial receptivity, implantation-related mechanisms, inflammatory signaling and potentially microbiome-mediated regulation of the uterine microenvironment [4,5,13]. In this scenario, the microbiome-associated functional changes identified in our study may represent an additional component of the biological alterations characterizing the fibroid-associated uterine milieu that can influence reproductive function.

This study has some limitations, including the relatively small sample size and the low-biomass nature of myometrial tissue, which requires caution when interpreting microbiome findings. In addition, BMI, dietary habits, hormonal status, and concomitant medication or antibiotic use were not systematically available and therefore could not be incorporated as covariates, representing potential sources of residual confounding. An extraction blank was processed in parallel with the tissue samples and yielded no retained reads after bioinformatic filtering; however, PCR and sequencing controls were not included. Hence, contamination introduced during amplification or sequencing, as well as residual reagent- or laboratory-derived contamination, cannot be completely excluded [22,24]. Accordingly, the observed taxonomic profiles should be interpreted cautiously. This limitation may also affect downstream PICRUSt2-based functional predictions, which depend on the underlying taxonomic profiles, as well as the microbiome–metabolite–cytokine correlation analyses. These integrative analyses should therefore be considered exploratory and hypothesis-generating rather than evidence of direct microbial–metabolic or microbial–immune interactions.

Finally, the study did not include direct fertility outcome measures, such as implantation rates, pregnancy outcomes, or clinical endometrial receptivity. Thus, the proposed links between the observed microbial, metabolic, and inflammatory signatures and implantation or endometrial receptivity remain hypothetical and require additional validation in studies including direct reproductive endpoints.

Furthermore, functional profiles were inferred from 16S rRNA gene sequencing using PICRUSt2 and represent predicted microbial functional potential rather than directly measured microbial activity or metabolite production.

Future studies incorporating fluorescence in situ hybridization (FISH) to independently confirm and localize bacterial signals in myometrial tissue, comprehensive negative-control designs (including extraction, PCR, and sequencing controls), and shotgun metagenomics, metatranscriptomics, and targeted myometrial metabolomics will be required to validate these findings and determine if the observed functional signatures reflect genuine microbial activity. Despite these limitations, by integrating, for the first time, predicted microbial functional alterations, systemic metabolic signatures and microbiome–immune–lipid interaction networks, our data support the existence of a context-dependent microbiome–immune–metabolic axis in UF biology. Future longitudinal studies integrating uterine microbiome profiling with endometrial receptivity markers and clinical reproductive outcomes will be essential to determine whether these signatures represent causal contributors, modulators or consequences of fibroid-associated reproductive dysfunction.

## Figures and Tables

**Table 1:** Clinical and demographic characteristics of the study population. Data are expressed as median (IQR) or frequency (%).

Patients’ Characteristics	All Patients (n = 21)
Age, years, median (IQR) [min–max]	48 (14) [18–73]
Heavy menstrual bleeding, n (%)	15 (71.4%)
Adenomyosis, n (%)	7 (33.3%)
Subserosal myoma, n (%)	10 (47.6%)
Pain, n (%)	18 (85.7%)
Multiple fibromatosis, n (%)	13 (61.9%)
Intramural myoma, n (%)	19 (90.5%)

## Data Availability

The data presented in this study are deposited in the NCBI Gene Expression Omnibus (GEO) repository, accession number GSE316107.

## Supplementary Materials

The following supporting information can be downloaded at: https://www.mdpi.com/article/10.3390/biology15171498/s1, Figure S1: Microbial diversity and community structure stratified by heavy menstrual bleeding status; Figure S2: Microbial diversity and community structure stratified by subserosal myoma status; Figure S3: Serum fatty acid profiles in uterine fibroid patients stratified by heavy menstrual bleeding status; Figure S4: Serum fatty acid profiles in uterine fibroid patients stratified by subserosal myoma status. Figure S5: Correlation networks between serum metabolites, cytokines, and tissue microbiota in uterine fibroid patients without heavy menstrual bleeding; Figure S6: Correlation networks between serum metabolites, cytokines, and tissue microbiota in uterine fibroid patients without subserosal myoma; Figure S7: Correlation networks between serum metabolites, cytokines, and tissue microbiota in uterine fibroid patients with subserosal myoma; Table S1: Differentially abundant predicted microbial functions between UF patients with and without heavy menstrual bleeding; Table S2: Differentially abundant predicted microbial functions between UF patients with and without subserosal myoma; Table S3: Serum cytokine levels in UF patients stratified by heavy menstrual bleeding status; Table S4: Serum cytokine levels in UF patients stratified by subserosal myoma status; Table S5: Serum cytokine levels in UF patients stratified by subserosal myoma status.

## Abbreviations

The following abbreviations are used in this manuscript:

UF	Uterine fibroid
MM	Myometrial microbiome
HM	Healthy myometrium
FFAs	Free fatty acids
SCFAs	Short-chain fatty acids
MCFAs	Medium-chain fatty acids
LCFAs	Long-chain fatty acids
GM	Gut microbiome
16S rRNA	16S ribosomal RNA
IQR	Interquartile range
GC-MS	Gas chromatography–mass spectrometry
ASVs	Amplicon sequence variants
DA	Differential abundance
PCoA	Principal coordinate analysis
PERMANOVA	Permutational multivariate analysis of variance
FDR	False discovery rate
HMB	Heavy menstrual bleeding
IL-1β	Interleukin-1 beta
IL-12p70	Interleukin-12p70
IL-18	Interleukin-18
IL-10	Interleukin-10
IL-6	Interleukin-6
TNFα	Tumor necrosis factor alpha
CCL2	C-C motif chemokine ligand 2
OPG	Osteoprotegerin
LBP	Lipopolysaccharide-binding protein
HC	Healthy control
