# Supplementary material for: Myometrial Microbiome in Uterine Fibroids: Functional Dysbiosis Versus Limited Taxonomic Change
Source: Biology (Basel). 2026 Sep 2;15(17):1498. doi: 10.3390/biology15171498 (PMC13564883; doi:10.3390/biology15171498)
Supplement: Supplementary file 1 [file biology-15-01498-s001.zip › biology-4472709-supplementary.pdf]

Supplementary materials

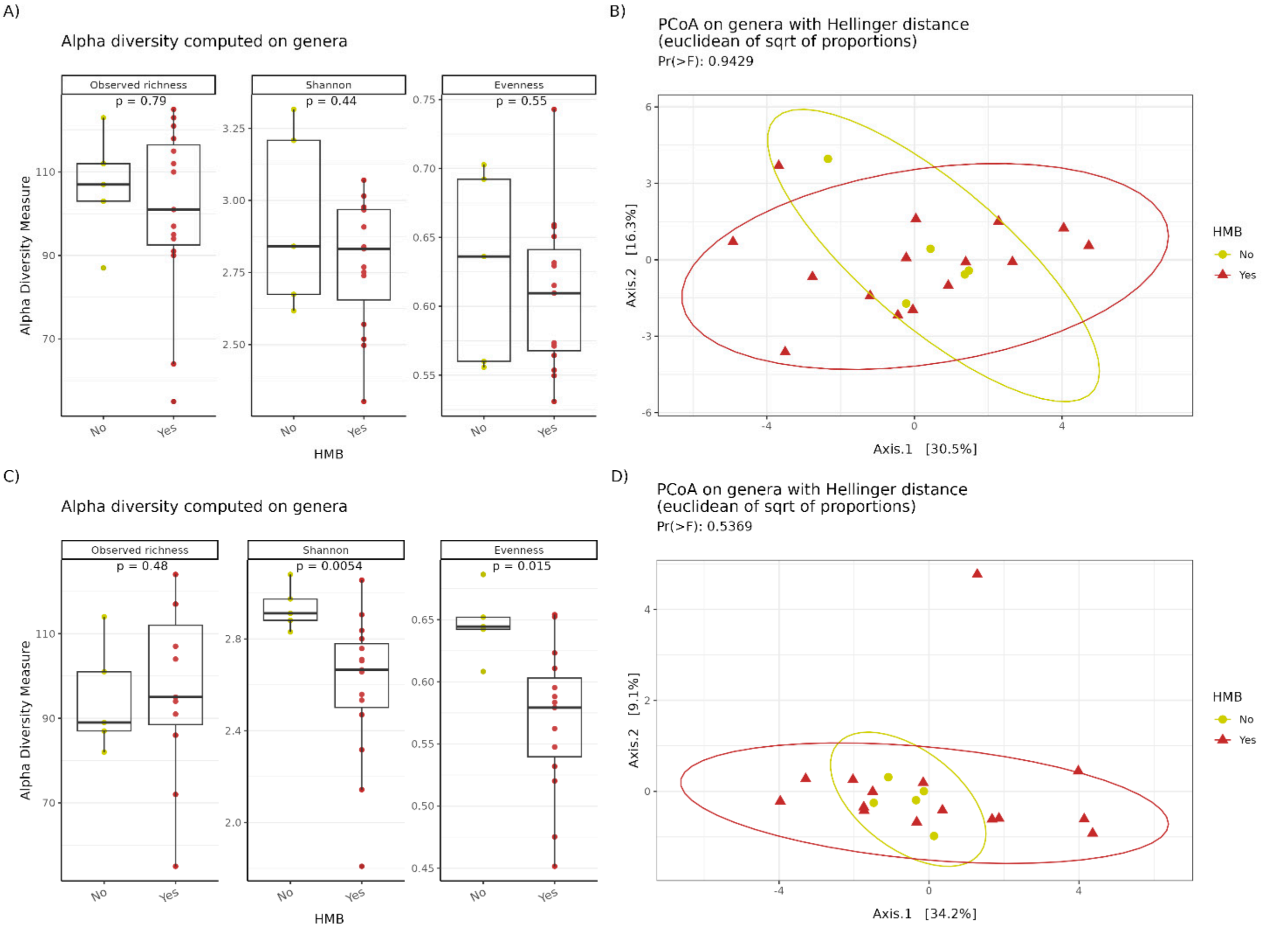

Supplementary Figure S1. Microbial diversity and community structure stratified by heavy menstrual bleeding status. (A, C) Box plots showing alpha diversity metrics (observed genus richness, Shannon index, and Pielou's evenness) in healthy myometrium **(A)** and fibroid uterine samples **(C)** from UF patients stratified by the presence or absence of heavy menstrual bleeding (HMB). Statistical differences were evaluated using the Mann–Whitney test; *p*-values < 0.05 were considered statistically significant. **(B, D)** Principal coordinate analysis (PCoA) based on Hellinger distances of genus-level transformed data in healthy myometrium **(B)** and fibroid uterine samples **(D)**, grouped by HMB status.

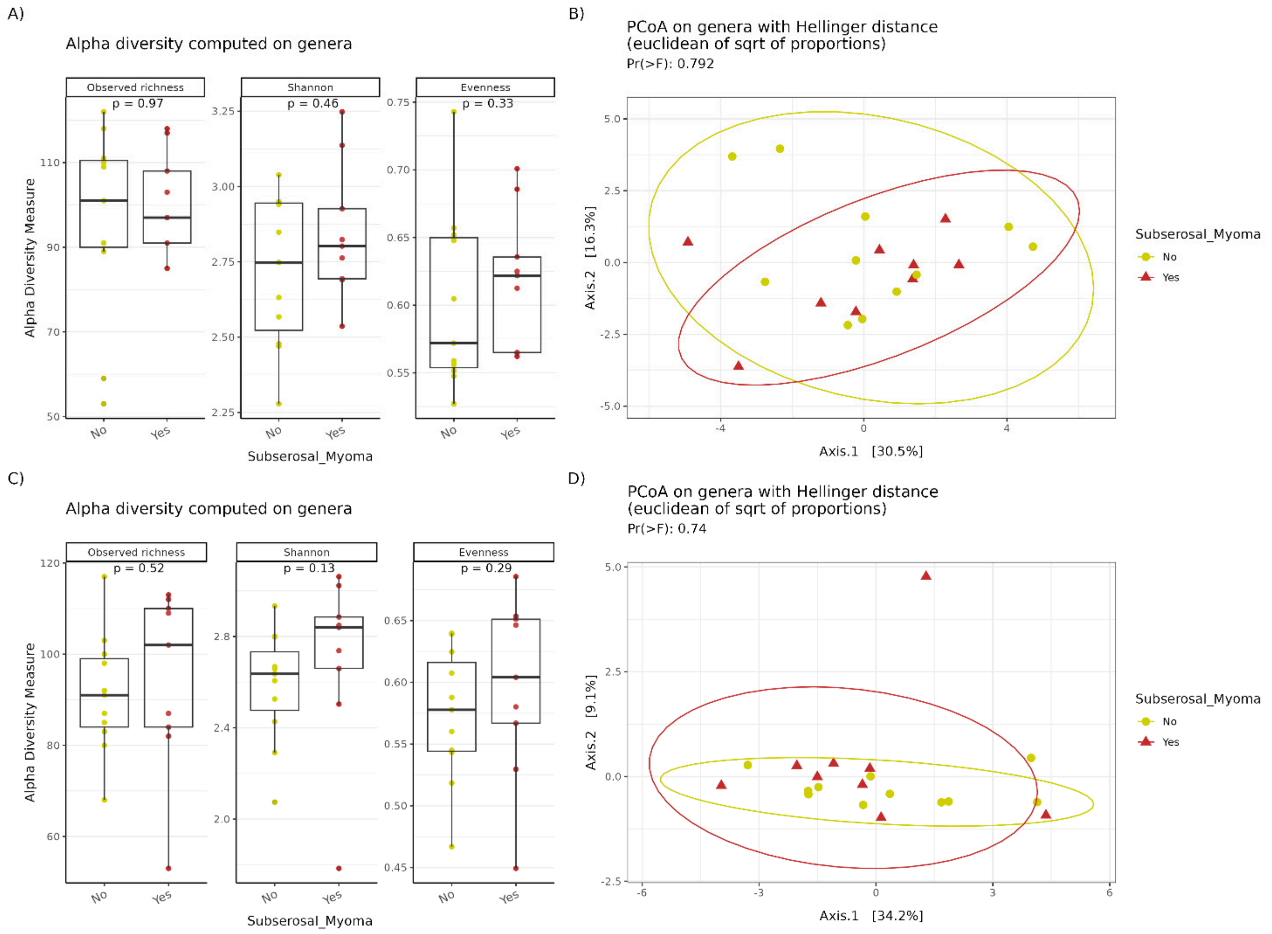

Supplementary Figure S2 . Microbial diversity and community structure stratified by subserosal myoma status. Box plots showing alpha diversity indices (observed genus richness, Shannon index, and Pielou's evenness) in healthy myometrium **(A)** and fibroid uterine samples **(C)** from UF patients stratified by the presence of a subserosal myoma. Statistical differences were assessed using the Mann–Whitney test; *p*-values < 0.05 were considered statistically significant. **(B, D)** Principal coordinate analysis (PCoA) based on Hellinger distance of transformed genus-level abundances in healthy myometrium **(B)** and fibroid uterine samples **(D)**, grouped according to subserosal myoma status.

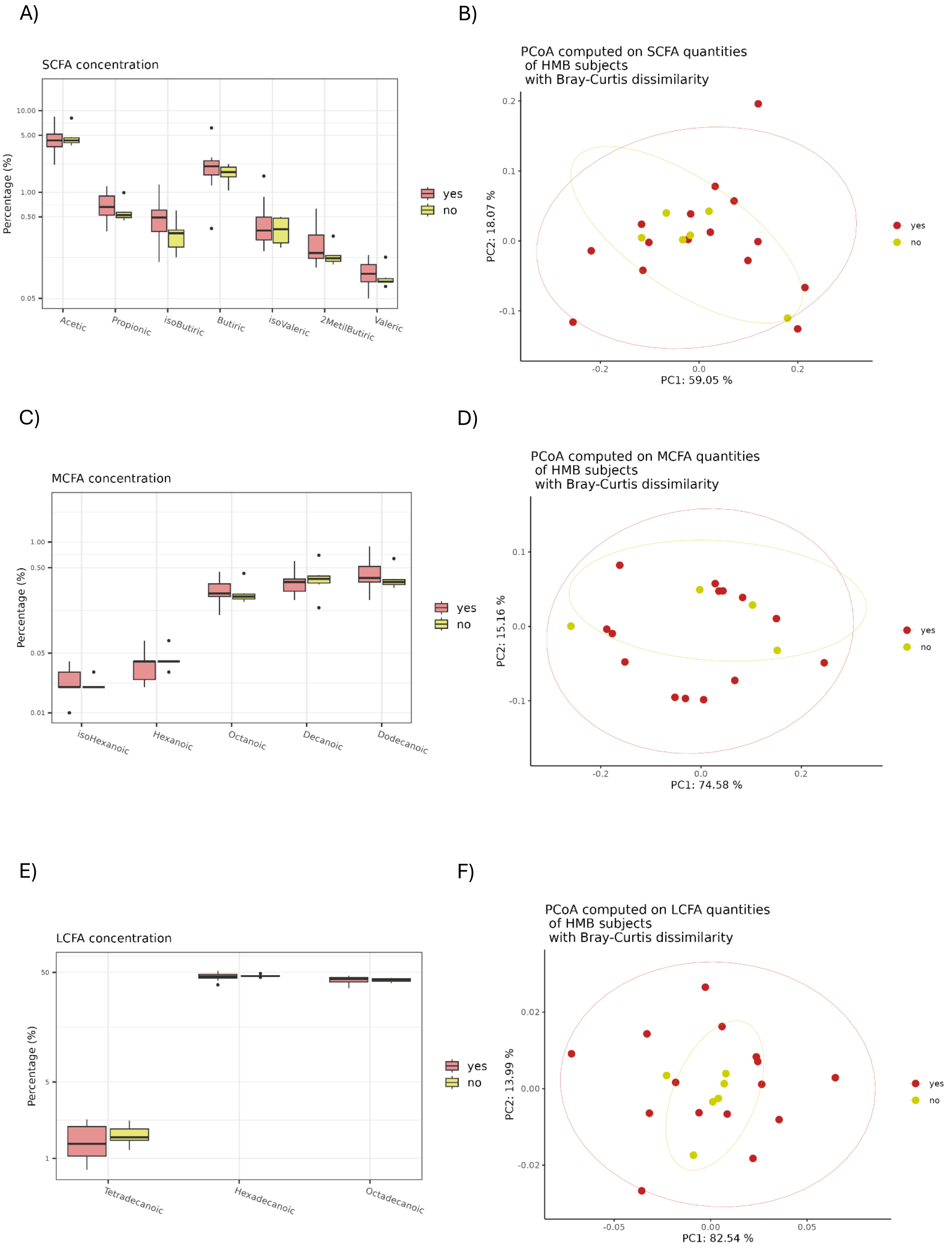

A)
SCFA concentration
Percentage (%)
10.00
5.00
1.00
0.50
0.05
Acetic
Propionic
isoButiric
Butiric
isoValeric
2MetilButiric
Valeric
yes
no
B)
PCoA computed on SCFA quantities
of HMB subjects
with Bray-Curtis dissimilarity
PC2: 18.07 %
PC1: 59.05 %
0.2
0.1
0.0
-0.1
-0.2
0.0
0.2
yes
no
C)
MCFA concentration
Percentage (%)
1.00
0.50
0.05
0.01
isoHexanoic
Hexanoic
Octanoic
Decanoic
Dodecanoic
yes
no
D)
PCoA computed on MCFA quantities
of HMB subjects
with Bray-Curtis dissimilarity
PC2: 15.16 %
PC1: 74.58 %
0.1
0.0
-0.1
-0.2
0.0
0.2
yes
no
E)
LCFA concentration
Percentage (%)
50
5
1
Tetradecanoic
Hexadecanoic
Octadecanoic
yes
no
F)
PCoA computed on LCFA quantities
of HMB subjects
with Bray-Curtis dissimilarity
PC2: 13.99 %
PC1: 82.54 %
0.02
0.00
-0.02
-0.05
0.00
0.05
yes
no

Supplementary Figure S3. **Serum fatty acid profiles in uterine fibroid patients stratified by heavy menstrual bleeding status**. Serum short-chain fatty acids (SCFAs), medium-chain fatty acids (MCFAs) and long-chain fatty acids (LCFAs) in patients with uterine fibroids (UF) stratified according to the presence of heavy menstrual bleeding (HMB). Box plots show the relative serum concentrations (%) of SCFAs (**A**), MCFAs (*C*) and LCFAs (**E**) in UF patients with (yes) and without (no) HMB. Principal coordinate analysis (PCoA) based on the Bray–Curtis dissimilarity index was used to explore potential differences in SCFA (**B**), MCFA (**D**) and LCFA (**F**) profiles between the two groups; PERMANOVA did not detect significant separation ($p > 0.05$). Group comparisons for individual FFAs were performed using the Mann–Whitney test with Benjamini–Hochberg correction for multiple testing; asterisks (*) indicate adjusted p-values < 0.05.

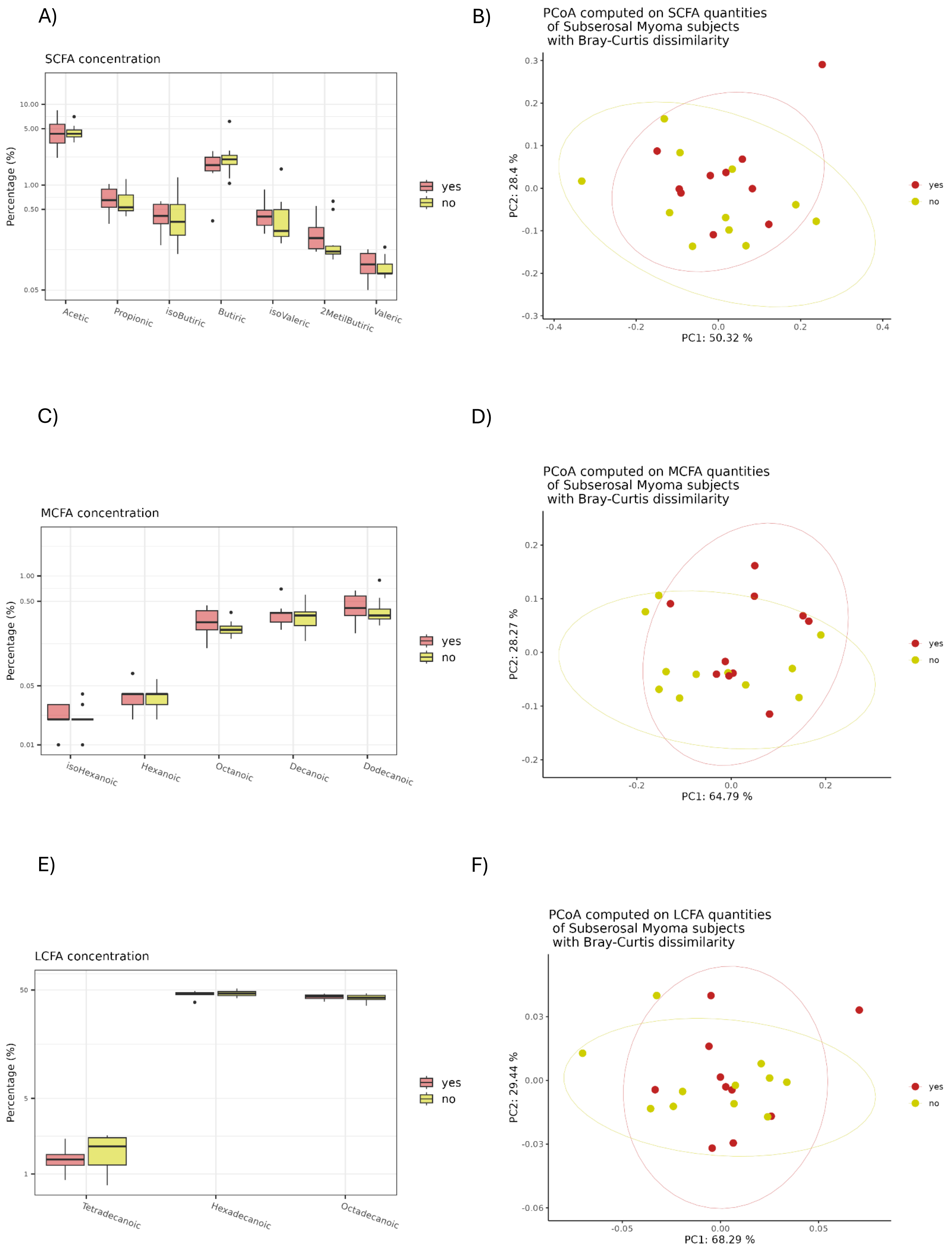
A)
SCFA concentration
Percentage (%)
10.00
5.00
1.00
0.50
0.05
Acetic
Propionic
isoButiric
Butiric
isoValeric
2MetilButiric
Valeric
yes
no
B)
PCoA computed on SCFA quantities
of Subserosal Myoma subjects
with Bray-Curtis dissimilarity
PC2: 28.4 %
PC1: 50.32 %
0.3
0.2
0.1
0.0
-0.1
-0.2
-0.3
-0.4
-0.2
0.0
0.2
0.4
yes
no
C)
MCFA concentration
Percentage (%)
1.00
0.50
0.05
0.01
isoHexanoic
Hexanoic
Octanoic
Decanoic
Dodecanoic
yes
no
D)
PCoA computed on MCFA quantities
of Subserosal Myoma subjects
with Bray-Curtis dissimilarity
PC2: 28.27 %
PC1: 64.79 %
0.2
0.1
0.0
-0.1
-0.2
-0.2
0.0
0.2
yes
no
E)
LCFA concentration
Percentage (%)
50
5
1
Tetradecanoic
Hexadecanoic
Octadecanoic
yes
no
F)
PCoA computed on LCFA quantities
of Subserosal Myoma subjects
with Bray-Curtis dissimilarity
PC2: 29.44 %
PC1: 68.29 %
0.03
0.00
-0.03
-0.06
-0.05
0.00
0.05
yes
no

Supplementary Figure S4. **Serum fatty acid profiles in uterine fibroid patients stratified by subserosal myoma status**. Serum short-chain fatty acids (SCFAs), medium-chain fatty acids (MCFAs) and long-chain fatty acids (LCFAs) in patients with uterine fibroids (UF) stratified according to the presence of subserosal myoma. Box plots show the relative serum concentrations (%) of SCFAs (**A**), MCFAs (**C**) and LCFAs (**E**) in UF patients with (yes) and without (no) subserosal myoma. Principal coordinate analysis (PCoA) based on the Bray–Curtis dissimilarity index was used to explore potential differences in SCFA (**B**), MCFA (**D**) and LCFA (**F**) profiles between the two groups; PERMANOVA did not detect significant separation ($p > 0.05$). Group comparisons for individual FFAs were performed using the Mann–Whitney test with Benjamini–Hochberg correction for multiple testing; asterisks (*) indicate adjusted p-values < 0.05.

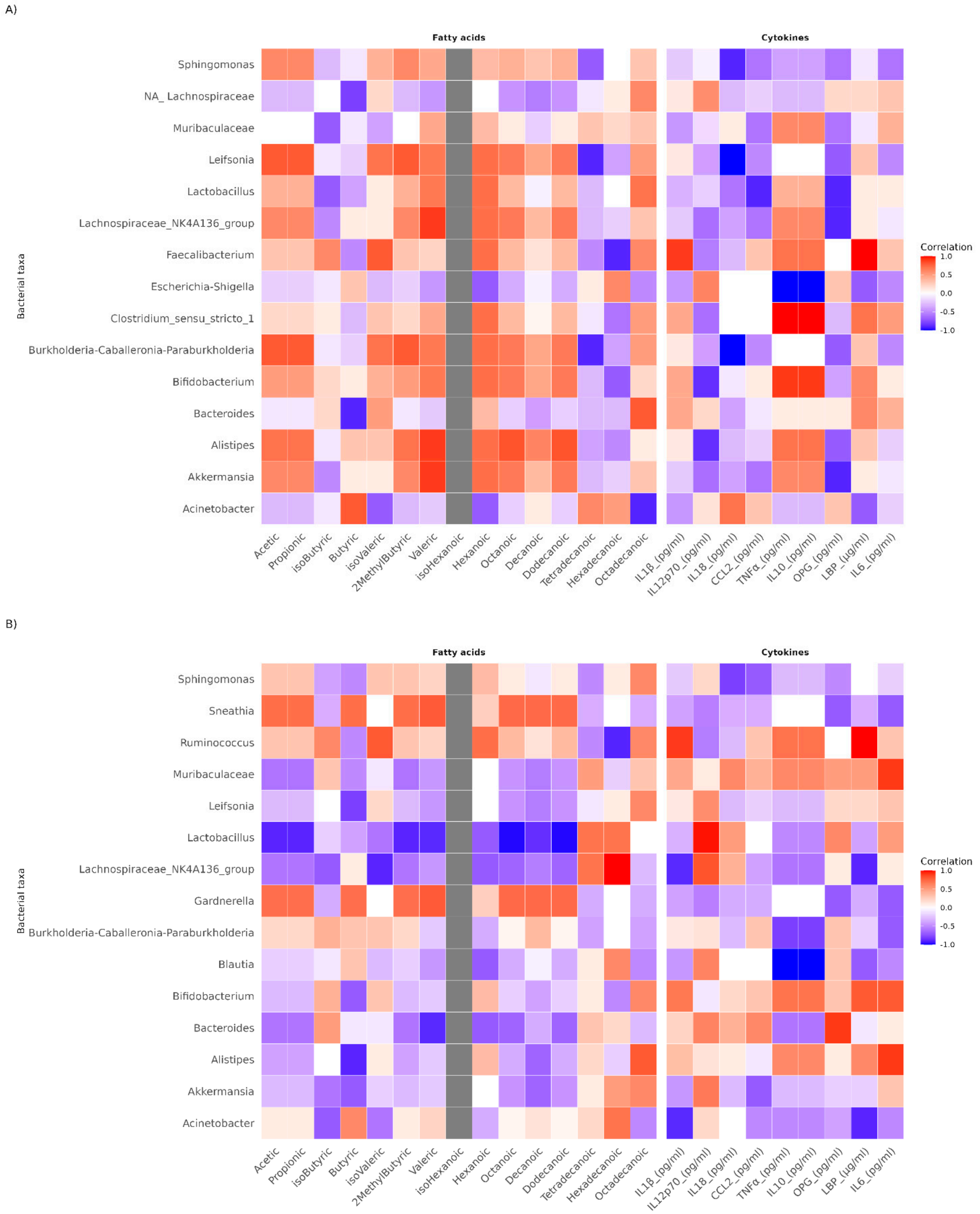

Supplementary Figure S5. Correlation networks between serum metabolites, cytokines, and tissue microbiota in uterine fibroid patients without heavy menstrual bleeding. Heatmaps of Spearman correlations between serum free fatty acids (FFAs), circulating cytokines and tissue microbial taxa in patients with uterine fibroids (UF) but without heavy menstrual bleeding (HMB). Panel (**A**) shows correlations for samples associated with healthy myometrium (HM), whereas panel (**B**) shows correlations for samples associated with uterine fibroid (UF). Red shades indicate positive

correlations, whereas blue shades indicate negative correlations; colour intensity reflects the strength of the correlation. Correlations were computed using Spearman's rank method. Asterisks (*) denote statistically significant correlations after multiple-testing correction (*padj* < 0.05).

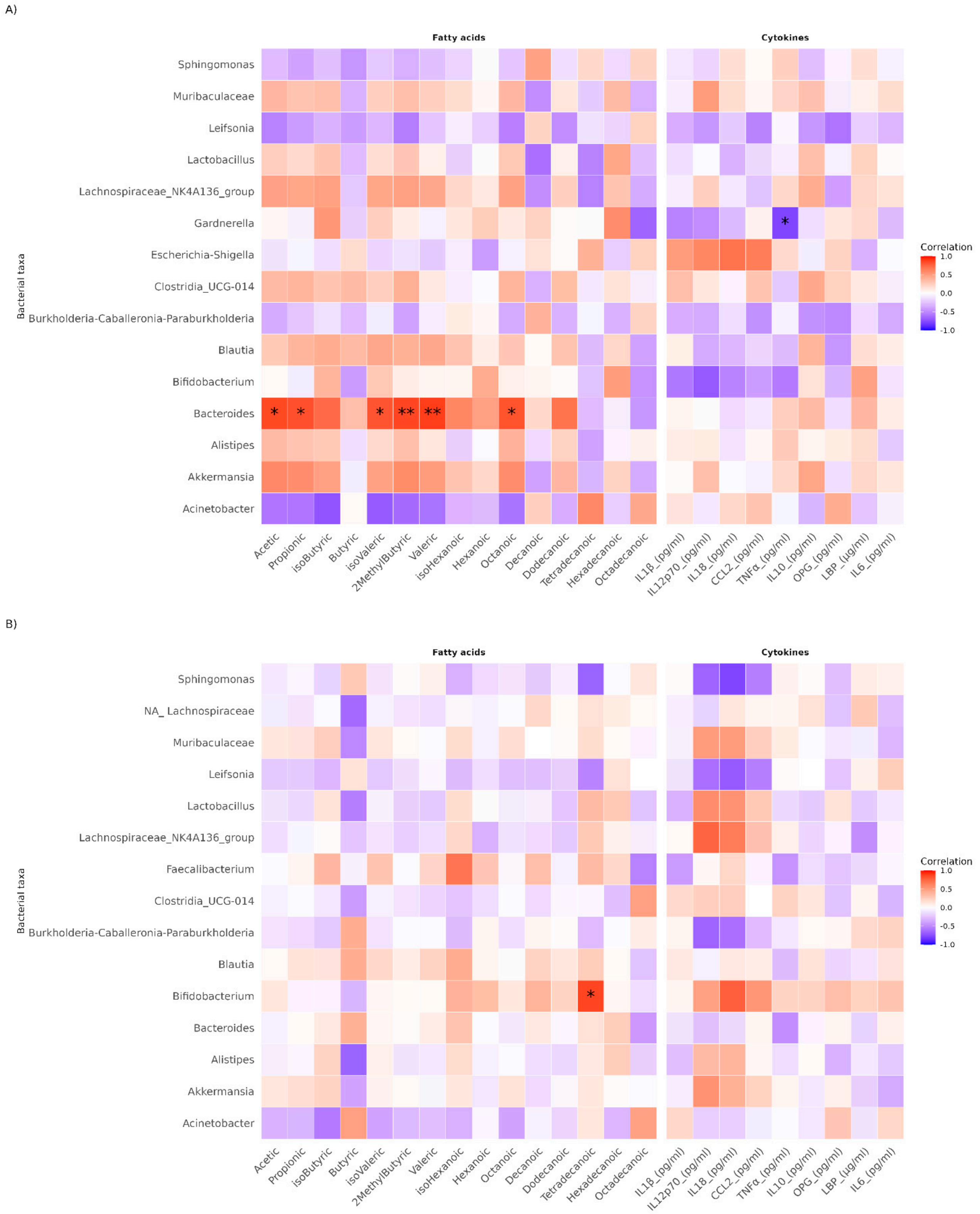

Supplementary Figure S6. Correlation networks between serum metabolites, cytokines, and tissue microbiota in uterine fibroid patients without subserosal myoma. Heatmaps of Spearman correlations between serum free fatty acids (FFAs), circulating cytokines and tissue microbial taxa in patients with uterine fibroids (UF) without subserosal myoma. Panel (**A**) shows correlations for samples associated with healthy myometrium (HM), whereas panel (**B**) shows correlations for samples associated with fibroid uterine (UF). Red shades indicate positive correlations, whereas blue

shades indicate negative correlations; colour intensity reflects the strength of the correlation. Correlations were computed using Spearman's rank method. Asterisks (*) denote statistically significant correlations after multiple-testing correction ($padj < 0.05$).

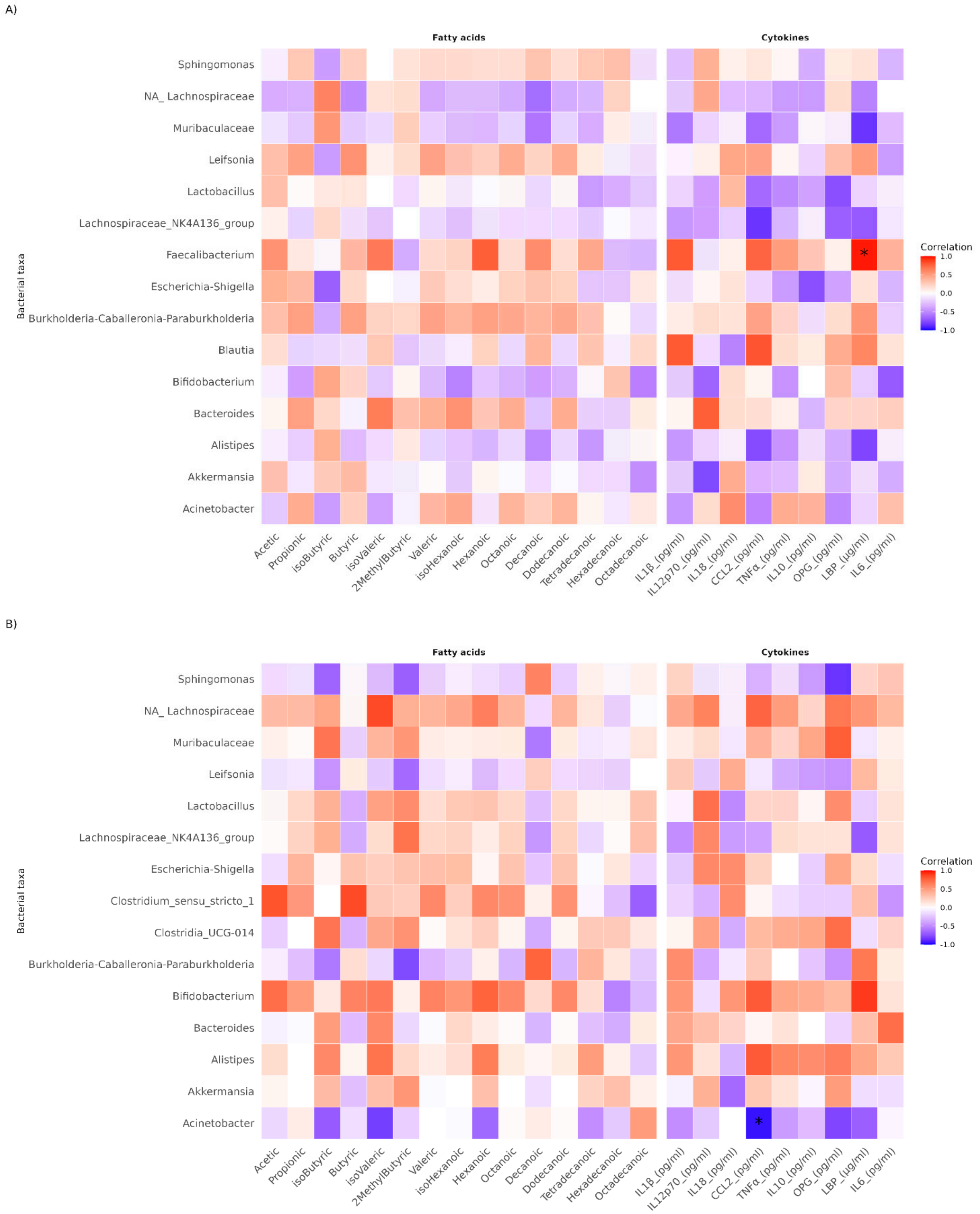

Supplementary Figure S7. Correlation networks between serum metabolites, cytokines, and tissue microbiota in uterine fibroid patients with subserosal myoma. Heatmaps of Spearman correlations between serum free fatty acids (FFAs), circulating cytokines and tissue microbial taxa in patients with uterine fibroids (UF) with subserosal myoma. Panel (**A**) shows correlations for samples associated with healthy myometrium (HM), whereas panel (**B**) shows correlations for samples associated with uterine fibroid (UF). Red shades indicate positive correlations, whereas blue shades

indicate negative correlations; colour intensity mirrors the strength of the correlation. Correlations were computed using Spearman's rank method. Asterisks (*) denote statistically significant correlations after multiple-testing correction (*padj* < 0.05).

**MetaCyc ID**	**Group with highest mean**	**logLDA score**	**p-value**
adenosylcobalamin biosynthesis I (early cobalt insertion)	HM	2.01	0.04
UDP-N-acetyl-D-glucosamine biosynthesis I	HM	2.01	0.02
polyisoprenoid biosynthesis (E. coli)	HM	2.02	0.05
superpathway of tetrahydrofolate biosynthesis and salvage	HM	2.03	0.01
cob(II)yrinate a,c-diamide biosynthesis I (early cobalt insertion)	HM	2.04	0.03
gondoate biosynthesis (anaerobic)	HM	2.06	0.04
L-1,2-propanediol degradation	HM	2.07	0.01
superpathway of L-tyrosine biosynthesis	UF	2.09	0.01
phosphatidylglycerol biosynthesis II (non-plastidic)	HM	2.10	0.05
phosphatidylglycerol biosynthesis I (plastidic)	HM	2.10	0.05
urate biosynthesis/inosine 5'-phosphate degradation	UF	2.13	0.02
lactose and galactose degradation I	HM	2.14	0.05
5-aminoimidazole ribonucleotide biosynthesis I	HM	2.14	0.01
L-arginine biosynthesis III (via N-acetyl-L-citrulline)	UF	2.15	0.02
inosine-5'-phosphate biosynthesis I	HM	2.15	0.02
gluconeogenesis I	HM	2.16	0.03
L-lysine biosynthesis II	HM	2.16	0.02
GDP-mannose biosynthesis	UF	2.16	0.001
dTDP-L-rhamnose biosynthesis I	UF	2.17	0.001
5-aminoimidazole ribonucleotide biosynthesis II	HM	2.18	0.01
superpathway of 5-aminoimidazole ribonucleotide biosynthesis	HM	2.18	0.01
superpathway of branched amino acid biosynthesis	UF	2.19	0.001
pyrimidine deoxyribonucleotides de novo biosynthesis II	HM	2.19	0.02
superpathway of L-threonine biosynthesis	HM	2.21	0.01
phosphopantothenate biosynthesis I	UF	2.23	0.01
L-ornithine biosynthesis	UF	2.23	0.05
pentose phosphate pathway	UF	2.25	0.01
UDP-N-acetylmuramoyl-pentapeptide biosynthesis II (lysine-containing)	HM	2.25	0.03
superpathway of thiamin diphosphate biosynthesis II	HM	2.26	0.03
Calvin-Benson-Bassham cycle	HM	2.26	0.05

4-hydroxyphenylacetate degradation	UF	2.27	0.01
coenzyme A biosynthesis I	HM	2.28	0.04
superpathway of adenosine nucleotides de novo biosynthesis I	HM	2.29	0.01
peptidoglycan biosynthesis III (mycobacteria)	HM	2.30	0.03
D-galacturonate degradation I	UF	2.30	0.001
ketogluconate metabolism	UF	2.31	0.01
tRNA charging	HM	2.31	0.03
L-isoleucine biosynthesis II	UF	2.31	0.01
UDP-N-acetylmuramoyl-pentapeptide biosynthesis I (meso-diaminopimelate containing)	HM	2.31	0.03
peptidoglycan biosynthesis I (meso-diaminopimelate containing)	HM	2.31	0.03
superpathway of histidine, purine, and pyrimidine biosynthesis	HM	2.32	0.03
chlorosalicylate degradation	UF	2.33	0.01
D-fructuronate degradation	UF	2.34	0.001
superpathway of GDP-mannose-derived O-antigen building blocks biosynthesis	UF	2.34	0.001
superpathway of salicylate degradation	UF	2.35	0.04
guanosine ribonucleotides de novo biosynthesis	HM	2.35	0.03
superpathway of polyamine biosynthesis I	UF	2.36	0.01
3-phenylpropanoate degradation	UF	2.37	0.01
D-galactarate degradation I	UF	2.38	0.01
superpathway of D-glucarate and D-galactarate degradation	UF	2.38	0.01
L-arabinose degradation IV	UF	2.41	0.01
L-histidine degradation II	UF	2.41	0.01
nicotinate degradation I	UF	2.41	0.01
GDP-D-glycero-α-D-manno-heptose biosynthesis	UF	2.42	0.001
protocatechuate degradation II (ortho-cleavage pathway)	UF	2.42	0.02
superpathway of 2,3-butanediol biosynthesis	UF	2.42	0.03
D-glucarate degradation I	UF	2.45	0.01
myo-inositol degradation I	UF	2.46	0.02
L-isoleucine biosynthesis I (from threonine)	UF	2.46	0.01
L-valine biosynthesis	UF	2.46	0.01
superpathway of L-aspartate and L-asparagine biosynthesis	HM	2.48	0.05
adenosine nucleotides degradation II	UF	2.63	0.01
myo-, chiro- and scillo-inositol degradation	UF	2.65	0.01
superpathway of (R,R)-butanediol biosynthesis	UF	2.68	0.01
L-leucine degradation I	UF	2.71	0.01
pyruvate fermentation to isobutanol (engineered)	UF	2.71	0.01
aromatic biogenic amine degradation (bacteria)	UF	2.78	0.01
aerobic respiration I (cytochrome c)	UF	2.86	0.02

Supplementary Table S1. Differentially abundant predicted microbial functions between healthy myometrium (HM) and uterine fibroid tissue (UF). Predicted microbial functions significantly differing between paired healthy myometrium (HM) and uterine fibroid (UF) tissue from the same patients. The table shows MetaCyc pathway IDs, the tissue with the highest mean abundance, the corresponding LDA scores, and p-values.

A)

MetaCyc ID	Group with highest mean (HMB/non-HMB)	LogLDA score	p-value
dTDP-L-rhamnose biosynthesis I	Yes	2.10	0.04
L-arginine biosynthesis IV (archaebacteria)	Yes	2.15	0.01
L-arginine biosynthesis I (via L-ornithine)	Yes	2.16	0.01
superpathway of glucose and xylose degradation	No	2.26	0.01
peptidoglycan maturation (meso-diaminopimelate containing)	Yes	2.31	0.04

B)

MetaCyc ID	Group with highest mean (HMB/non-HMB)	LogLDA score	p-value
dTDP-L-rhamnose biosynthesis I	Yes	2.12	0.04
L-arginine biosynthesis IV (archaebacteria)	Yes	2.13	0.01
L-arginine biosynthesis I (via L-ornithine)	Yes	2.12	0.01
superpathway of glucose and xylose degradation	No	2.24	0.01
peptidoglycan maturation (meso-diaminopimelate containing)	Yes	2.26	0.04

Supplementary Table S2. Differentially abundant predicted microbial functions between UF patients with and without heavy menstrual bleeding. Predicted microbial functions significantly differing between UF patients with and without HMB in healthy myometrium (**A**) and fibroid (**B**) tissue. The Table shows MetaCyc pathway IDs, the group with the highest mean abundance, the corresponding LDA scores, and p-values.

A)

MetaCyc ID	Group with highest mean (subserosal myoma/non-subserosal myoma)	LogLDA score	p-value
thiamin salvage II	No	2.08	0.04
D-galacturonate degradation I	No	2.11	0.02
superpathway of menaquinol-8 biosynthesis II	Yes	2.11	0.007
dTDP-L-rhamnose biosynthesis I	No	2.13	0.004
peptidoglycan biosynthesis V (lactam resistance)	Yes	2.18	0.01
cob(II)yrinate a,c-diamide biosynthesis I (early cobalt insertion)	Yes	2.19	0.007
GDP-mannose biosynthesis	No	2.20	0.02
L-glutamate and L-glutamine biosynthesis	Yes	2.21	0.02

B)

MetaCyc ID	Group with highest mean (subserosal myoma/non-subserosal myoma)	LogLDA score	p-value
thiamin salvage II	No	2.08	0.04

D-galacturonate degradation I	No	2.13	0.02
superpathway of menaquinol-8 biosynthesis II	Yes	2.08	0.007
dTDP-L-rhamnose biosynthesis I	No	2.14	0.004
peptidoglycan biosynthesis V (lactam resistance)	Yes	2.78	0.01
cob(II)yrinate a,c-diamide biosynthesis I (early cobalt insertion)	Yes	2.17	0.007
GDP-mannose biosynthesis	No	2.24	0.02
L-glutamate and L-glutamine biosynthesis	Yes	2.25	0.02

Supplementary Table S3. Differentially abundant predicted microbial functions between UF patients with and without subserosal myoma. Predicted microbial functions significantly differing between UF patients with and without subserosal myoma in healthy myometrium (**A**) and fibroid (**B**) tissue. The Table shows MetaCyc pathway IDs, the group with the highest mean abundance, the corresponding LDA scores, and p-values.

Cytokine	HMB (yes) median (IQR)	HMB (no) median (IQR)	p.value	p.adj (BH)
IL1β (pg/ml)	0.00 (0-0)	0.22 (0-0.29)	0.2	0.86
IL12p70 (pg/ml)	0 (0-0.36)	0.13 (0-0.2)	0.91	1
IL18 (pg/ml)	174 (128-226)	194 (191-244)	0.92	1
CCL2 (pg/ml)	268 (257-299)	307 (294-405)	0.2	0.86
TNFα (pg/ml)	6.92 (6.33-9.86)	8.18 (7.96-11.40)	0.76	1
IL10 (pg/ml)	1.44 (1.13-1.94)	1.74 (1.12-1.97)	0.85	1
OPG (pg/ml)	1020 (861-1120)	921 (910-1044)	0.77	1
LBP (µg/ml)	10.87 (7.22-12.17)	11.74 (6.87-14.11)	1	1
IL6 (pg/ml)	1.86 (1.48-5.39)	2.94 (2-5.96)	0.28	0.86

Supplementary Table S4. Serum cytokine levels in UF patients stratified by heavy menstrual bleeding status. Comparison of serum cytokine levels between UF patients with and without heavy menstrual bleeding (HMB). Differences between groups were assessed using the Wilcoxon rank-sum test. Data are reported as median and interquartile range (IQR).

Cytokine	Subserosal myoma (yes) median (IQR)	Subserosal myoma (no) median (IQR)	p.value	p.adj (BH)
IL1β (pg/ml)	0 (0-0.22)	0.00 (0.00-0.07)	0.49	0.9
IL12p70 (pg/ml)	0.13 (0-0.36)	0.00 (0.00-0.21)	0.85	0.93
IL18 (pg/ml)	200 (191-223)	188 (147-244)	0.86	0.93
CCL2 (pg/ml)	299 (294-307)	267 (250-405)	0.05	0.41
TNFα (pg/ml)	8.18 (6.92-8.55)	6.92 (5.30-9.86)	0.26	0.84
IL10 (pg/ml)	1.74 (1.36-1.94)	1.44 (1.11-1.53)	0.37	0.84
OPG (pg/ml)	941 (853-1044)	1054 (861-1184)	0.72	0.93
LBP (µg/ml)	8.61 (7.73-12.30)	11.28 (9.93-14.90)	0.37	0.84
IL6 (pg/ml)	2.44 (2-5.96)	3.31 (1.59-6.59)	0.93	0.93

Supplementary Table S5. **Serum cytokine levels in UF patients stratified by subserosal myoma status**. Comparison of serum cytokine levels between UF patients with and without subserosal myoma. Differences between groups were assessed using the Wilcoxon rank-sum test. Data are reported as median and interquartile range (IQR).
